# Epigenomic Control of Thermogenic Adipocyte Differentiation and Function

**DOI:** 10.3390/ijms19061793

**Published:** 2018-06-17

**Authors:** Xu Peng, Qiongyi Zhang, Cheng Liao, Weiping Han, Feng Xu

**Affiliations:** 1 Institute of Molecular and Cell Biology, Agency for Science, Technology and Research (A*STAR), 61 Biopolis Drive, Singapore 138673, Singapore; xpeng@imcb.a-star.edu.sg (X.P.); Zhang_Qiongyi@sbic.a-star.edu.sg (Q.Z.); 2 Laboratory of Metabolic Medicine, Singapore Bioimaging Consortium, A*STAR, 11 Biopolis Way, Singapore 138667, Singapore; Weiping_Han@sbic.a-star.edu.sg; 3 Department of Preclinical Development, Translation Medicine and External Research, Jiangsu Hengrui Medicine Co., Ltd., 778 Dongfang Road, Shanghai 200085, China; liaocheng@shhrp.com; 4 Department of Biochemistry, Yong Loo Lin School of Medicine, National University of Singapore, 8 Medical Drive, Singapore 117596, Singapore

**Keywords:** epigenome, thermogenesis, adipocyte, histone modification, DNA methylation

## Abstract

Obesity and its associated metabolic disorders are spreading at a fast pace throughout the world; thus, effective therapeutic approaches are necessary to combat this epidemic. Obesity develops when there is a greater caloric intake than energy expenditure. Promoting energy expenditure has recently attracted much attention as a promising approach for the management of body weight. Thermogenic adipocytes are capable of burning fat to dissipate chemical energy into heat, thereby enhancing energy expenditure. After the recent re-discovery of thermogenic adipocytes in adult humans, much effort has focused on understanding the molecular mechanisms, especially the epigenetic mechanisms, which regulate thermogenic adipocyte development and function. A number of chromatin signatures, such as histone modifications, DNA methylation, chromatin accessibilities, and interactions, have been profiled at the genome level and analyzed in various murine and human thermogenic fat cell systems. Moreover, writers and erasers, as well as readers of the epigenome are also investigated using genomic tools in thermogenic adipocytes. In this review, we summarize and discuss the recent advance in these studies and highlight the insights gained into the epigenomic regulation of thermogenic program as well as the pathogenesis of human metabolic diseases.

## 1. Introduction

Adipose tissue plays a key role in the control of metabolic homeostasis in mammals. As the main component and functional unit of adipose tissue, adipocytes store lipids, respond to insulin stimulation, and also, secrete various metabolic regulatory hormones known as adipokines. Based on their origin, morphology, and function, adipocytes can be classified into three different types: (1) classic white adipocytes that store excess energy in the form of triglycerides; (2) classic brown adipocytes derived from a myf-5 positive muscle-like cellular lineage [1] that specialize in burning fat to produce heat to counteract hypothermia; and (3) “brown-like” adipocytes that are derived from both myf-5 negative and positive lineages [2] and reside within white adipose depots. “Brown-like” adipocytes are also referred as “beige” or “brite” adipocytes [3,4,5]. Although beige adipocytes are thermogenic and express high levels of UCP1 (uncoupling protein 1, a marker gene of brown fat), their general gene expression pattern is distinct from the patterns of brown and white fat [4]. A common feature of these thermogenic fat cells is that they contain a high density of mitochondria and are capable of dissipating electrochemical energy through proton leak to generate heat. During this process, UCP1 plays a key role as the proton transporter. UCP1-positive BAT (brown adipose tissue) has been previously identified only in infants and rodents. It was long considered absent in adult humans. However, in 2009, several independent groups reported the identification of brown-like fat depots in healthy adults using PET-CT (Positron emission tomography-computed tomography) scans or human biopsies [6,7,8,9]. These discoveries attracted significant interest in harnessing the thermogenic activity of BAT in obesity management and spurred intensive studies of brown fat biology and energy metabolism. Multiple lines of evidence suggest that increased thermogenic BAT activity has beneficial effects on whole body metabolic homeostasis [7,9,10]. For example, BAT transplantation improves glucose homeostasis and insulin sensitivity in obese mice [11]. In contrast, the ablation of BAT by surgical removal or genetic approaches leads to obese phenotype in rodents [12,13,14]. In addition, *Ucp1* knock-out also results in decreased energy metabolism and the onset of obesity [15]. Therefore, the promotion of BAT recruitment and activity has great potential in the treatment of metabolic disorders and has attracted much attention in the metabolism field in recent years.

Fat cells are originally derived from multipotent MSCs (mesenchymal stem cells) which can give rise to various cell types in response to appropriate environmental cues. The differentiation of fat cells is a complex physiological process that requires the concerted regulation of gene expression through numerous adipogenic factors. Among these factors, PPARγ (peroxisome proliferator activated receptor γ) plays a central role by controlling the expression of a whole panel of adipogenic genes during the differentiation process [16]. In addition, many histone-modifying enzymes and chromatin remodeling factors are among the adipogenic regulators, suggesting that epigenetic mechanisms play essential roles in controlling adipogenesis [17]. Indeed, significant adipogenic and thermogenic marker genes, such as adiponectin, leptin and the *Ucp1*, are under the control of bivalent histone marks H3 K4 and K27 tri-methylation [18]. In addition, inhibiting DNA methylation using 5-azacytidine in C3H10T1/2 MSCs commits these cells to the adipocyte lineage [19]. To gain a fundamental understanding of the gene regulation networks that control the development of thermogenic adipocytes, it is necessary to systematically profile the epigenome and define the *cis*-regulatory elements, such as enhancers, that modulate adipogenic and thermogenic gene expression. In recent years, these profiling works have been greatly facilitated by the discovery of signature histone modifications for these *cis*-elements [20,21,22,23] and the development of the ChIP-seq (chromatin Immunoprecipitation-sequencing) technique. Moreover, a number of transcription factors intervene with the transcriptional network and crosstalk with the readers and writers of the epigenome to fine tune adipogenic gene expression. Through computational analyses of the chromatin landscape, many *trans*-regulatory factors of the thermogenic program have been identified. In this review, we highlight the recent progress in epigenomic studies of thermogenic adipocytes. In addition, we summarize the *trans*-regulatory factors identified through the analysis of epigenomic profiles. Finally, we discuss the involvement of epigenomic regulation mechanisms in metabolic diseases. Due to space limitation, we will focus on genome-wide studies of histone modifications, DNA methylation, and chromatin remodeling in the context of thermogenic adipocyte development and function. Gene-specific epigenetic regulation will not be covered in this review. miRNAs represent another major component of epigenetic regulation. However, studies on miRNAs in adipocyte signaling/thermogenesis will not be discussed in this review, because their regulatory mechanisms are largely gene-specific [24,25,26,27]. Those studies have been reviewed extensively elsewhere [28,29].

## 2. Genome-Wide Studies on Histone Modifications during Thermogenic Adipogenesis

**Figure 1:**
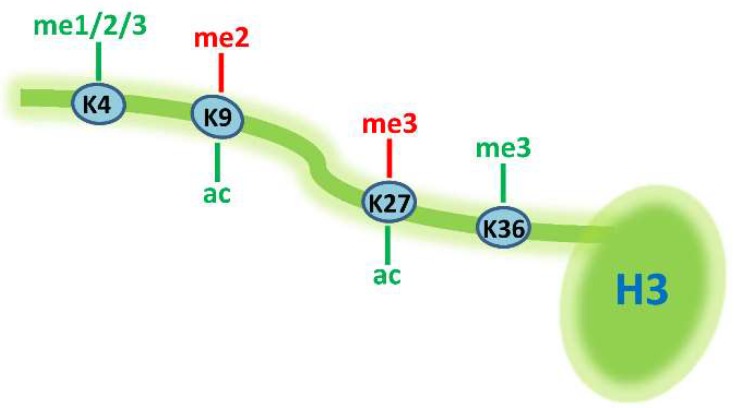
Histone modifications studied at the genome-wide level during thermogenic adipocyte differentiation. Permissive histone marks are colored in green, while repressive histone marks are in red.

Histones are the main component of chromatin and are targets of extensive post-translational modifications (PTM). These modifications include acetylation, methylation, phosphorylation, and many other newly discovered types of acylation [30] which constitute prominent epigenetic mechanisms in eukaryotic gene regulation. Through adding or removing numerous permissive or repressive histone marks, chromatin structure and gene expression are dynamically regulated during the differentiation of various mammalian cells, including adipocytes. So far, epigenomic studies, in the context of thermogenic adipocyte differentiation, have been mainly focused on the PTMs in the *N*-terminal tail of histone H3 (Figure 1). Depending on the site and type of modification, these PTMs can be either permissive or repressive to gene activity. For example, the three states of methylation on H3K4 (mono-methylation, me1; di-methylation, me2 and tri-methylation, me3) and acetylation on H3K9 and H3K27 are generally linked to gene activation; while methylation on H3K9 and H3K27 are repressive to gene expression; moreover, H3K36me3 is a marker for transcriptional elongation. To identify chromatin signatures for thermogenic genes in brown adipocytes, Pan et al. found that a significant subset of BAT-selective genes are marked by H3K27me3 in pre-adipocytes, and the removal of this repressive chromatin mark by JMJD3 (Jumonji domain containing protein 3) is required for the expression of these genes in mature adipocytes [31]. Furthermore, a recent study by Brunmeir et al., through additional epigenomic profiling, showed that the removal of H3K27me3 is necessary, although not sufficient, to promote brown gene expression at the late stage of brown adipogenesis [32]. Instead, the pre-deposition of H3K4me1 at these brown genes is a key step for the subsequent activation of gene transcription in mature brown cells. In addition, this study also profiled the dynamic changes of H3K27ac, a chromatin marker of active enhancers, which enabled the systematic identification of stage-specific active enhancers during brown adipogenesis [32].

H3K4me1 is another chromatin marker that is enriched at the enhancers [20]. In the context of brown adipocyte differentiation, it was found that the methyltransferase MLL4 (mixed-lineage leukemia 4) is responsible for adding H3K4me1 to the enhancers for enhancer commissioning and subsequent enrichment of H3K27ac, mediator and RNA polymerase II for cell type-specific gene expression [33]. Recently, the same group further showed that the H3K4me1 methyltransferases MLL3/MLL4 are required for the binding of the H3K27ac acetyltransferases CBP (CREB-binding protein)/p300 at the enhancer elements and the establishment of the so-called super-enhancers in brown adipocytes [34]. Based on these results, the authors proposed that MLL3 and MLL4 are major enhancer epigenomic writers that are responsible for enhancer priming, and this process is followed by the activation of enhancers through H3K27 acetylation by CBP/p300 during brown cell differentiation. In contrast to MLL4, LSD1 (lysine-specific demethylase 1) is the demethylase responsible for the removal of H3K4me1/me2 markers from chromatin [35]. In BAT, LSD1 was found to be associated with PRDM16 (PR domain containing 16) to co-localize at white fat-selective genes and repress these genes through demethylation of H3K4me1/me2 at the promoters [36]. Moreover, in a very recent, elegant study that investigated the reprogramming of adipocyte cellular identity during temperature changes, H3K27ac and H3K4me1 were shown to be essential for the reprogramming of the thermogenic beige adipocytes [37]. In this report, the authors examined the changes in cellular identity upon warming for the two types of thermogenic adipocytes and found that beige but not brown adipocytes underwent a reprogramming from a brown to a white-like state. Through cell type-specific transcriptomic profiling and PCA (principal component analysis) analysis, it was shown that the transcriptome of cold-exposed beige adipocytes reassembles the transcriptome of brown cells, while the re-warmed beige adipocytes showed a global gene expression pattern similar to that of warm white adipocytes. Accompanying the transcriptomic reprogramming, massive remodeling of the chromatin landscape occurred when cold-exposed beige adipocytes were re-warmed. This was evidenced by a shift of 30% of the H3K27ac peaks between cold and warm states in beige adipocytes. Strikingly, only 0.7% of H3K27ac peaks showed differential enrichment between cold beige adipocytes and cold brown adipocytes, while even less H3K27ac peaks (0.2%) differed between warm beige adipocytes and warm white adipocytes. Intriguingly, re-warmed beige adipocytes retained an epigenomic memory of their prior cold exposure by poising a subset of enhancers through H3K4me1 at key thermogenic genes, including *Ucp1* and *Cpt1b* (carnitine palmitoyltransferase IB), thus enabling the rapid re-activation of these genes when exposed to cold again.

In addition to the abovementioned histone marks, H3K9me2 and its demethylase JMJD1A were also shown to be involved in the regulation of thermogenic programming [38]. In brown adipocytes, JMJD1A is phosphorylated by PKA (protein kinase A), and this phosphorylation event promotes the binding of JMJD1A to the SWI/SNF (SWItch/Sucrose Non-Fermentable) chromatin remodeling complex which further facilitates long-range chromatin interactions between enhancers and promoters of brown genes. In parallel, JMJD1A also serves a second role by removing H3K9me2 from brown genes to allow their long-term stable expression [38,39]. In a more recent study, the authors further dissected the roles of JMJD1A and H3K9me2 in regulating thermogenic gene expression upon acute or chronic cold stress. They found that in BAT, H3K9me2 is already at low levels at thermogenic genes such as *Ucp1*, therefore the acute activation of *Ucp1* does not require JMJD1A-mediated H3K9me2 demethylation. In contrast, during chronic cold exposure, the induction of thermogenic genes in beige fat requires the removal of H3K9me2 by JMJD1A from their enhancers/promoters, and this process is mediated by the β-adrenergic-dependent phosphorylation of S265 in JMJD1A. Besides the majority of epigenomic profiling that has been conducted in murine systems, Loft et al. performed a study involving H3K27ac ChIP-seq in hMADS (human multipotent adipose-derived stem) cells with or without their newly-discovered browning factor KLF11 (Kruppel-like factor 11). This work showed that H3K27ac positively correlates with KLF11 binding at the brite-selective genes, providing intriguing insights into thermogenic programming in human adipocytes [40].

## 3. Genome-Wide Studies on Chromatin Remodeling during Thermogenic Adipogenesis

Chromatin remodeling is another essential epigenetic mechanism required for gene regulation. During gene activation, chromatin structure must be opened up for the transcriptional machinery to pass through and chromatin remodeling is fundamentally involved in this process. In general, the open chromatin regions within the genome are mainly found at active promoters or *cis*-regulatory elements such as enhancers. A previous study applied the FAIRE-seq method (formaldehyde-assisted isolation of regulatory elements sequencing) to 3T3-L1 white adipocytes in the profiling of open chromatin regions during adipogenesis [41]. Recently, the same group further characterized open chromatin regions in BAT through the same approach [42]. From the BAT-specific FAIRE peaks, the authors identified an enrichment of the NF-1 binding motif. Subsequently, through both in vitro and in vivo functional studies, they showed that NFIA (nuclear factor I/A) binds to the brown-fat-specific enhancers and further facilitates the binding of PPARγ to promote chromatin opening and gene activation. In addition, the authors performed ATAC-seq (assays for transposase-accessible chromatin sequencing) for the profiling of accessible chromatin regions in brown adipocytes [42]. ATAC-seq was also used to unveil the functional mechanism of a newly discovered regulator of thermogenesis and energy expenditure, IL-10 (interleukin 10) [43]. In this study, the authors showed that the chromatin structure at the enhancer/promoter regions of thermogenic genes became more accessible in mature beige adipocytes and these chromatin architecture changes were largely blocked by IL-10, resulting in the reduction of thermogenic gene expression and further energy expenditure [43].

## 4. Genome-Wide Studies on DNA Methylation during Thermogenic Adipogenesis

DNA molecules can be methylated on cytosine and adenine, and cytosine methylation has been widely studied as an important epigenetic mechanism in gene repression. In the context of thermogenic gene expression and adipogenesis, DNA methylation has been studied in a gene-specific manner at the *Ucp1* promoter/enhancer, and the results showed that DNA methylation anti-correlates with *Ucp1* expression [44,45,46]. Recently, Lim et al. used the RRBS method (reduced representation bisulfite sequencing) to profile the dynamic changes in the DNA methylome during brown adipogenesis [47]. It was found that DNA methylation is relatively stable across different stages of adipogenesis, and a group of Hox (Homeobox) genes showed differential promoter methylation between white and brown adipogenesis. The epigenomic studies in the regulation of thermogenic programming are summarized below in Table 1.

## 5. Genome-Wide Studies on the Writers, Erasers and Readers of the Epigenome during Thermogenic Adipogenesis

**Figure 2:**
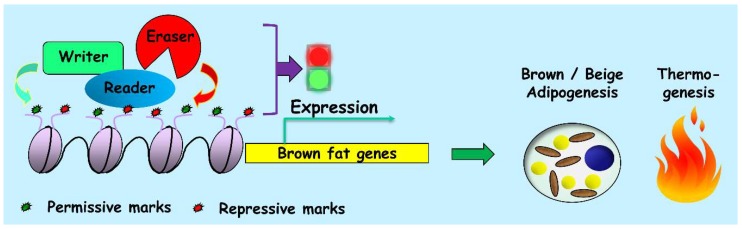
Schematic illustration of the collaborative regulation of thermogenic fat cell differentiation by writers, erasers, and readers of the epigenome.

Chromatin modifications are covalently added to the histones or DNA molecules by epigenetic writers, such as acetylases and methylases [53,54,55]. These epigenetic markers can also be removed by erasers like deacetylases and demethylases. In addition, various histone modifications are hypothesized to compose a “histone code” [56] which can be interpreted by the readers of the code harboring specific functional domains, such as bromodomain (reader of lysine acetylation) [57] and chromodomain (reader of lysine methylation) [58,59,60]. Given the fundamental involvement of epigenetic mechanisms in the regulation of thermogenic adipocyte development and function, it is not surprising that a number of epigenomic writers, erasers and readers have been observed actively participate in this process (Table 2 and Figure 2).

For example, H3K4me1 is a key chromatin marker for enhancer commissioning [33], and it was shown to be required for the priming of thermogenic genes for subsequent activation [32,37]. Genome-wide binding of its writer, MLL4, was profiled, and the data revealed that MLL4 binds preferentially to active enhancers together with lineage-determining TFs (transcription factors) in a cell-type-specific manner. Further, MLL4 defines the super-enhancers in brown adipocytes and is essential for the loading of the mediator and polymerase II on enhancers as well as subsequent thermogenic gene expression and cell differentiation [33,34]. On the other hand, the H3K4me1/2 eraser, LSD1, was reported to co-localize with PRDM16 at white fat-selective genes in BAT and repress their expression through H3K4me1/2 demethylation from the promoters [36], and possibly through the recruitment of the CoREST (Corepressor to RE1 silencing transcription factor) repressor complex [61]. Moreover, through analyzing the binding motifs in LSD1 peaks, the authors discovered that NRF1 (nuclear respiratory factor 1) is a binding partner for LSD1 and subsequently, showed that these coumpounds form a complex which co-localizes at the brown-selective gene promoters to activate their expression [61].

H3K27ac markers active enhancers and have been demonstrated as essential players in the regulation of thermogenic genes in brown and beige fat. The CBP/p300 acetylases are the writers for this permissive chromatin marker. Genome-wide profiling of CBP occupancy in brown adipocytes unveiled its role in promoting enhancer activation following enhancer-priming by MLL4 [34,49]. In addition, CBP binding identifies super-enhancers in murine brown adipocytes and is enriched at PPARγ-defined super-enhancers in hMADS cells, suggesting its pivotal role in promoting adipogenic as well as thermogenic gene activation [34,40].

H3K9me2 is a repressive chromatin marker that blocks gene transcription; therefore, it has to be removed to allow the activation of its decorated genes. JMJD1A is an eraser of H3K9me2, and it plays a dual role in the regulation of the thermogenic program [38]. On one hand, JMJD1A binds to the SWI/SNF chromatin remodeling complex to establish long-range chromatin interactions between the enhancers and promoters of brown genes. On the other hand, JMJD1A removes H3K9me2 from the enhancers/promoters of thermogenic genes to allow their induction upon chronic cold exposure in beige fat. Both of these processes are mediated by the β-adrenergic-dependent phosphorylation of JMJD1A at S265. Moreover, a genome-wide binding analysis in β-adrenergic stimulated brown adipocytes revealed that half of the PRDM16 binding sites overlap with those of JMJD1A, and those overlapping sites are found in several thermogenic genes [62].

BRD4 (Bromodomain-containing protein 4) is a bromodomain-containing protein that reads lysine acetylation. As an epigenomic reader, it binds to active enhancers and promoters [63,64] to control the induction of cell identity genes. Through genetic ablation in Myf5-positive progenitor cells of BAT and muscle lineages, *Brd4* was shown to be required for the expression of genes defining adipocyte and myocyte identity as well as for BAT and muscle development in vivo. Genome-wide profiling of BRD4 binding revealed that it binds to cell identity genes together with lineage-determining TFs at the active enhancers. Further mechanistic studies suggested a working model by which lineage-determining TFs coordinate with the H3K4me1 writers, MLL3/MLL4, and the H3K27ac writers, CBP/p300, to recruit BRD4, presumably through acetyl-lysine recognition, to the enhancers of lineage specific genes to activate their expression [65].

## 6. Novel Regulators of Thermogenic Adipogenesis Identified through Epigenomic Studies

Differential gene expression analysis was widely used for the identification of trans-regulators in various cell types in the early days. For instance, the master adipogenic regulator PPARγ is selectively expressed in fat and markedly upregulated during the course of adipogenesis [16], while the key thermogenic factor, UCP1, is specific to brown fat and can be further induced upon cold exposure. Recently, along with the advances in genomic techniques, especially deep-sequencing, genome-wide profiling, followed by computational analysis, has become an increasingly powerful tool in the search for novel regulators of given biological processes. By comparing differential PPARγ binding between WAT (white adipose tissue) and BAT and the subsequent TF motif search in BAT-specific PPARγ binding sites, EBF2 (early B cell factor-2) was identified as a key transcriptional regulator promoting brown fat development [66]. Through epigenomic studies, a number of novel regulators of thermogenic adipogenesis were identified (Table 3). Firstly, one group of novel regulators were discovered through a binding motif search in cell type-specific enhancer elements or regions enriched for certain chromatin markers. For example, by searching late stage brown cell specific enhancers, SIX1 (SIX homeobox 1) was identified as an activator of brown adipogenesis [32], while RREB1 (Ras-responsive element-binding protein 1) was found through searching a collection of H3K27me3 peaks at BAT-selective gene promoter regions for over-represented TF motifs [31]. (2) In recent years, super-enhancer association analysis has identified many novel regulators for thermogenic programming. The concept of a super-enhancer was introduced in 2013 as clusters of typical enhancers that are enriched for master regulators and mediator binding [67]. These super-enhancers can also be defined by the enrichment of certain chromatin markers, such as H3K27ac, and are “super” in size, transcription factor density, and ability to activate transcription [67]. Further analyses showed that the genes associated with super-enhancers are often the ones defining a cell’s identity or important for cellular functions. Using super-enhancer association analysis, KLF11 was identified as a novel factor required for rosiglitazone-induced browning in human brite adipocytes [40]. PIM1 (Pim-1 Proto-Oncogene, Serine/Threonine Kinase) and RREB1 were also shown to be associated with brown specific super-enhancers and further validated as activators of thermogenic program in murine brown adipocytes [32]. In addition, DLC1 (deleted in liver cancer 1) was found to be associated with super-enhancers in both white and brown adipocytes, and it was subsequently confirmed to be required for both adipogenesis and thermogenic activation [68]. Besides these protein factors, a microRNA regulator has also been discovered by super-enhancer association analysis. Based on its distance from eWAT (epididymal white adipose tissue) super-enhancers of all miRNAs associated with BAT super-enhancers, miR-32 was identified as one of the top candidates for thermogenic regulation. Indeed, it was further demonstrated to be essential for brown fat thermogenesis and subcutaneous white fat browning through driving FGF21 (fibroblast growth factor 21) expression and secretion from BAT [69]. (3) Chromatin structure and interaction can also be used for thermogenic regulator identification. The browning repressor, IRX3 (iroquois homeobox 3) was identified by analyzing FTO (fat mass and obesity-associated) gene long-range chromatin interactions [70,71], while the abovementioned thermogenic activator NFIA was found through analyzing brown specific open chromatin regions [42]. (4) Finally, DNA methylation profiling and a comparative bioinformatic analysis between white and brown adipocytes identified *Hoxc10* (Homeobox C10) as a gene that is hypomethylated and highly expressed in white fat [47] and represses brown markers in WAT [72].

## 7. Genome-Wide Studies on Chromatin Modifications and Interactions in Disease Models

Given the prominent roles for epigenetic mechanisms in the regulation of the development and function of fat storage and fat burning adipocytes, it is not surprising that a fundamental involvement of chromatin modifications and interactions in the pathogenesis of metabolic diseases has been observed. For example, insulin resistance is a hallmark of type II diabetes, and it also occurs at a high rate in obese and aging populations. Despite extensive investigation, the molecular mechanism leading to insulin resistance is still not completely understood. In this regard, a recent study focused on the epigenomic changes that might contribute to the pathogenesis of insulin resistance [73]. In the study, the authors profiled the epigenome consisting of H3K4me1, H3K4me3, H3K27ac, H3K27me3, H3K36me3, and H3K79me2 in the 3T3-L1 adipocytes which has a demonstrated phenotype of insulin resistance. Although no significant changes in the overall binding peaks for these chromatin modifications were identified, it was found through analyzing H3K27ac profiles that this modification was altered preferentially at distal enhancers after treatments inducing insulin resistance. Moreover, the binding motif for GRs (glucocorticoid receptors) is over-represented in upregulated H3K27ac peaks, indicating a functional role for GRs in the pathogenesis of insulin resistance. Through subsequent gain and loss-of-function studies, GRs have been validated as mediators of insulin resistance [73]. Another example for the involvement of epigenetic mechanisms in metabolic disorders comes from the *FTO* variants, where certain SNPs (single-nucleotide polymorphisms) at the intronic region of the *FTO* gene were found to be strongly associated with obesity in humans through large scale GWAS (genome-wide association studies). Early studies were focused on the role of the mRNA demethylase encoded by the *FTO* gene in the regulation of metabolic processes. But surprisingly, subsequent investigations revealed that the obesity-associated, non-coding region within the *FTO* gene actually serves as an enhancer element for the *IRX3* gene through long-range chromatin interactions with its promoter [70,71]. The obesity-associated, single nucleotide alteration enhances *IRX3* expression in beige adipocytes, leading to a shift from the energy-dissipating beige adipocytes to energy-storing white adipocytes [70,71]. Without the advanced 4C-seq (circular chromosome conformation capture sequencing) technique for the profiling of long-range chromatin interactions, this intriguing mechanism for obesity development would not have been discovered.

## 8. Concluding Remarks

There is little doubt that epigenetic mechanisms are fundamentally involved in the regulation of thermogenic gene expression programming, and the recent advances in genomic approaches have greatly enhanced our capability to understand the transcriptional regulatory network at the systematic level. Significant insights into the molecular mechanisms controlling thermogenic adipocyte development and function have been gained in the past a few years through epigenomic profiling and in-depth bioinformatic analyses. Given the great potential for harnessing BAT activity in the treatment of metabolic disorders, such as obesity and diabetes, the next step in our research should be applying the knowledge of thermogenic regulation to human diseases. For example, a number of trans-regulators that promote or suppress thermogenesis have been identified through the studies discussed in this review, and these factors could be used as potential drug targets to develop therapeutics for obesity management. Another direction to advance our understanding of the epigenomic regulation of thermogenesis would be to put the epigenomic profiles into a bigger picture by integrating multi-omics datasets to systematically determine the crosstalk among the epigenome, transcriptome, metabolome, and lipidome. These studies will provide further insights into the pathogenesis of metabolic diseases and open new avenues for therapeutic interventions.

## Figures and Tables

**Table 1:** Epigenomic profiling of histone modifications, the open chromatin region and DNA methylation in thermogenic adipocytes and fat tissues.

Chromatin Markers/Regions	Function	Systems	Refs
H3K4me1	Enhancer priming	Immortalized primary brown pre-adipocytes; C3H10T1/2 mesenchymal stem cells (MSCs); brown adipose tissue (BAT); FACS sorted cell type-specific nuclei from cold/warm beige and brown adipocytes	[32,33,34,36,37,48,49,50]
H3K4me2	Gene activation	Immortalized primary brown pre-adipocytes; BAT	[33,34,36]
H3K4me3	Promoter activation	Immortalized primary brown preadipocytes; C3H10T1/2 MSCs; BAT	[32,33,34,38,48,51]
H3K9me2	Gene repression	Immortalized primary brown pre-adipocytes;	[34]
H3K27me3	Gene repression	Immortalized primary brown pre-adipocytes; C3H10T1/2 MSCs;	[31,32,34]
H3K36me3	Transcriptional elongation	Immortalized primary brown pre-adipocytes;	[34]
H3K9ac	Gene activation	C3H10T1/2 MSCs	[32]
H3K27ac	Enhancer activation	Immortalized primary brown pre-adipocytes; BAT; C3H10T1/2 MSCs; FACS sorted cell type-specific nuclei from cold/warm beige and brown adipocytes; human multipotent adipose-derived stem (hMADS) cells	[32,33,34,37,38,40,42,48,49,50,51,52]
Open chromatin region (FAIRE-seq (formaldehyde-assisted isolation of regulatory elements sequencing) and ATAC-seq (assays for transposase-accessible chromatin sequencing))	Active promoters and enhancers	Immortalized primary brown preadipocytes; BAT; Immortalized beige pre-adipocytes	[34,42,43]
DNA methylation	Gene repression	Primary brown pre-adipocytes	[47]

Abbreviations used in the table: MSC (Mesenchymal stem cell); BAT (Brown adipose tissue); FACS (Fluorescence-activated cell sorting); hMADS (Human multipotent adipose-derived stem); FAIRE-seq (Formaldehyde-assisted isolation of regulatory elements-sequencing); ATAC-seq (Assays for transposase-accessible chromatin-sequencing).

**Table 2:** Genome-wide profiling of the writers, erasers, and readers of the epigenome in thermogenic adipocytes and fat tissues.

Chromatin Factors	Function	Systems	Refs
MLL4 (mixed-lineage leukemia 4)	H3K4me1/2 methylase (writer)	Immortalized primary brown pre-adipocytes	[33,34]
LSD1 (lysine-specific demethylase 1)	H3K4me1/2 demethylase (eraser)	Brown adipocytes; BAT	[36,61]
JMJD1A (Jumonji domain containing 3)	H3K9me2 demethylase (eraser)	Immortalized primary brown pre-adipocytes	[38]
CBP (Carnitine palmitoyltransferase)	H3K27ac acetylase (writer)	Immortalized primary brown pre-adipocytes; hMADS cells	[34,40,49]
BRD4	Reader of lysine acetylation	Immortalized primary brown pre-adipocytes	[65]

Abbreviations used in the table: BAT (Brown adipose tissue); hMADS (Human multipotent adipose-derived stem); MLL4 (Mixed-lineage leukemia 4); LSD1 (Lysine-specific demethylase 1); JMJD1A (Jumonji domain containing protein 1A); CBP (CREB-binding protein); BRD4 (Bromodomain-containing protein 4).

**Table 3:** Novel regulators of thermogenic adipogenesis identified through epigenomic studies.

Regulators	Function	Approaches	Refs
**SIX1**	Promotes brown adipogenesis	Binding motif search in enhancers	[32]
**RREB1**	Promotes brown adipogenesis	Binding motif search in H3K27me3 peaks; Super-enhancer association analysis	[31,32]
**KLF11**	Promotes browning in human brite adipocytes	Super-enhancer association analysis	[40]
**PIM1**	Promotes brown adipogenesis	Super-enhancer association analysis	[32]
miR-32	Promotes brown fat thermogenesis and white fat browning	Super-enhancer association analysis	[69]
**IRX3**	Represses white fat browning	Long-range chromatin interaction analysis	[70,71]
**NFIA**	Promotes brown adipogenesis	Open chromatin region analysis	[42]
**HOXC10**	Represses white fat browning	DNA methylation analysis	[47]

Abbreviations used in the table: SIX1 (SIX Homeobox 1); RREB1 (Ras-responsive element-binding protein 1); KLF11 (Kruppel-like factor 11); PIM1 (Pim-1 Proto-Oncogene, Serine/Threonine Kinase); IRX3 (Iroquois homeobox 3); NFIA (Nuclear factor I/A); HOXC10 (Homeobox C10).

## Abbreviations

BAT	Brown adipose tissue
WAT	White adipose tissue
eWAT	Epididymal white adipose tissue
PET-CT	Positron emission tomography-computed tomography
MSC	Mesenchymal stem cell
PTM	Post-translational modification
PCA	Principal component analysis
hMADS	Human multipotent adipose-derived stem
ChIP-seq	Chromatin immunoprecipitation-sequencing
FAIRE-seq	Formaldehyde-assisted isolation of regulatory elements-sequencing
ATAC-seq	Assays for transposase-accessible chromatin-sequencing
4C-seq	Circular chromosome conformation capture-sequencing
RRBS	Reduced representation bisulfite sequencing
FACS	Fluorescence-activated cell sorting
SNP	Single-nucleotide polymorphism
GWAS	Genome-wide association study
TF	Transcription factor
UCP1	Uncoupling protein 1
PPARγ	Peroxisome Proliferator Activated Receptor Gamma
EBF2	Early B cell factor-2
HOXC10	Homeobox C10
CBP	CREB-Binding protein
MLL4	Mixed-lineage leukemia 4
PRDM16	PR domain containing 16
CPT1b	Carnitine palmitoyltransferase IB
NFIA	Nuclear factor I/A
IL-10	Interleukin 10
KLF11	Kruppel-like factor 11
LSD1	Lysine-specific demethylase 1
IRX3	Iroquois homeobox 3
JMJD3	Jumonji domain containing protein 3
SWI/SNF	SWItch/sucrose non-fermentable
BRD4	Bromodomain-containing protein 4
PKA	Protein kinase A
GR	Glucocorticoid receptor
SIX1	SIX Homeobox 1
RREB1	Ras-responsive element-binding protein 1
PIM1	Pim-1 Proto-Oncogene, Serine/Threonine Kinase
DLC1	Deleted in liver cancer 1
FTO	Fat mass and obesity-associated
FGF21	Fibroblast growth factor 21
CoREST	Co-repressor to RE1 silencing transcription factor
NRF1	Nuclear respiratory factor 1

## References

[1] Seale P., Bjork B., Yang W., Kajimura S., Chin S., Kuang S., Scime A., Devarakonda S., Conroe H.M., Erdjument-Bromage H. (2008). PRDM16 controls a brown fat/skeletal muscle switch. Nature.

[2] Sanchez-Gurmaches J., Guertin D.A. (2014). Adipocytes arise from multiple lineages that are heterogeneously and dynamically distributed. Nat. Commun..

[3] Peirce V., Carobbio S., Vidal-Puig A. (2014). The different shades of fat. Nature.

[4] Wu J., Bostrom P., Sparks L.M., Ye L., Choi J.H., Giang A.H., Khandekar M., Virtanen K.A., Nuutila P., Schaart G. (2012). Beige adipocytes are a distinct type of thermogenic fat cell in mouse and human. Cell.

[5] Ikeda K., Maretich P., Kajimura S. (2018). The common and distinct features of brown and beige adipocytes. Trends Endocrinol. Metab..

[6] Van Marken Lichtenbelt W.D., Vanhommerig J.W., Smulders N.M., Drossaerts J.M., Kemerink G.J., Bouvy N.D., Schrauwen P., Teule G.J. (2009). Cold-activated brown adipose tissue in healthy men. N. Engl. J. Med..

[7] Cypess A.M., Lehman S., Williams G., Tal I., Rodman D., Goldfine A.B., Kuo F.C., Palmer E.L., Tseng Y.H., Doria A. (2009). Identification and importance of brown adipose tissue in adult humans. N. Engl. J. Med..

[8] Virtanen K.A., Lidell M.E., Orava J., Heglind M., Westergren R., Niemi T., Taittonen M., Laine J., Savisto N.J., Enerback S. (2009). Functional brown adipose tissue in healthy adults. N. Engl. J. Med..

[9] Saito M., Okamatsu-Ogura Y., Matsushita M., Watanabe K., Yoneshiro T., Nio-Kobayashi J., Iwanaga T., Miyagawa M., Kameya T., Nakada K. (2009). High incidence of metabolically active brown adipose tissue in healthy adult humans: Effects of cold exposure and adiposity. Diabetes.

[10] Bartelt A., Bruns O.T., Reimer R., Hohenberg H., Ittrich H., Peldschus K., Kaul M.G., Tromsdorf U.I., Weller H., Waurisch C. (2011). Brown adipose tissue activity controls triglyceride clearance. Nat. Med..

[11] Stanford K.I., Middelbeek R.J., Townsend K.L., An D., Nygaard E.B., Hitchcox K.M., Markan K.R., Nakano K., Hirshman M.F., Tseng Y.H. (2013). Brown adipose tissue regulates glucose homeostasis and insulin sensitivity. J. Clin. Investig..

[12] Connolly E., Morrisey R.D., Carnie J.A. (1982). The effect of interscapular brown adipose tissue removal on body-weight and cold response in the mouse. Br. J. Nutr..

[13] Hamann A., Flier J.S., Lowell B.B. (1996). Decreased brown fat markedly enhances susceptibility to diet-induced obesity, diabetes, and hyperlipidemia. Endocrinology.

[14] Lowell B.B., V S.S., Hamann A., Lawitts J.A., Himms-Hagen J., Boyer B.B., Kozak L.P., Flier J.S. (1993). Development of obesity in transgenic mice after genetic ablation of brown adipose tissue. Nature.

[15] Feldmann H.M., Golozoubova V., Cannon B., Nedergaard J. (2009). Ucp1 ablation induces obesity and abolishes diet-induced thermogenesis in mice exempt from thermal stress by living at thermoneutrality. Cell Metab..

[16] Tontonoz P., Hu E., Spiegelman B.M. (1994). Stimulation of adipogenesis in fibroblasts by ppar gamma 2, a lipid-activated transcription factor. Cell.

[17] Farmer S.R. (2006). Transcriptional control of adipocyte formation. Cell Metab..

[18] Bracken A.P., Dietrich N., Pasini D., Hansen K.H., Helin K. (2006). Genome-wide mapping of polycomb target genes unravels their roles in cell fate transitions. Genes Dev..

[19] Bowers R.R., Kim J.W., Otto T.C., Lane M.D. (2006). Stable stem cell commitment to the adipocyte lineage by inhibition of DNA methylation: Role of the BMP-4 gene. Proc. Natl. Acad. Sci. USA.

[20] Heintzman N.D., Hon G.C., Hawkins R.D., Kheradpour P., Stark A., Harp L.F., Ye Z., Lee L.K., Stuart R.K., Ching C.W. (2009). Histone modifications at human enhancers reflect global cell-type-specific gene expression. Nature.

[21] Heintzman N.D., Stuart R.K., Hon G., Fu Y., Ching C.W., Hawkins R.D., Barrera L.O., Van Calcar S., Qu C., Ching K.A. (2007). Distinct and predictive chromatin signatures of transcriptional promoters and enhancers in the human genome. Nat. Genet..

[22] Rada-Iglesias A., Bajpai R., Swigut T., Brugmann S.A., Flynn R.A., Wysocka J. (2011). A unique chromatin signature uncovers early developmental enhancers in humans. Nature.

[23] Creyghton M.P., Cheng A.W., Welstead G.G., Kooistra T., Carey B.W., Steine E.J., Hanna J., Lodato M.A., Frampton G.M., Sharp P.A. (2010). Histone H3K27ac separates active from poised enhancers and predicts developmental state. Proc. Natl. Acad. Sci. USA.

[24] Sun L., Xie H., Mori M.A., Alexander R., Yuan B., Hattangadi S.M., Liu Q., Kahn C.R., Lodish H.F. (2011). Mir193b-365 is essential for brown fat differentiation. Nat. Cell Biol..

[25] Adaikalakoteswari A., Vatish M., Alam M.T., Ott S., Kumar S., Saravanan P. (2017). Low vitamin B12 in pregnancy is associated with adipose-derived circulating mirs targeting ppargamma and insulin resistance. J. Clin. Endocrinol. Metab..

[26] Oldenburg A., Briand N., Sorensen A.L., Cahyani I., Shah A., Moskaug J.O., Collas P. (2017). A lipodystrophy-causing lamin a mutant alters conformation and epigenetic regulation of the anti-adipogenic MIR335 locus. J. Cell Biol..

[27] Nardelli C., Iaffaldano L., Pilone V., Labruna G., Ferrigno M., Carlomagno N., Dodaro C.A., Forestieri P., Buono P., Salvatore F. (2017). Changes in the microrna profile observed in the subcutaneous adipose tissue of obese patients after laparoscopic adjustable gastric banding. J. Obes..

[28] Alexander R., Lodish H., Sun L. (2011). Micrornas in adipogenesis and as therapeutic targets for obesity. Expert Opin. Ther. Targets.

[29] Shamsi F., Zhang H., Tseng Y.H. (2017). Microrna regulation of brown adipogenesis and thermogenic energy expenditure. Front. Endocrinol..

[30] Huang H., Sabari B.R., Garcia B.A., Allis C.D., Zhao Y. (2014). Snapshot: Histone modifications. Cell.

[31] Pan D., Huang L., Zhu L.J., Zou T., Ou J., Zhou W., Wang Y.X. (2015). Jmjd3-mediated H3K27me3 dynamics orchestrate brown fat development and regulate white fat plasticity. Dev. Cell.

[32] Brunmeir R., Wu J., Peng X., Kim S.Y., Julien S.G., Zhang Q., Xie W., Xu F. (2016). Comparative transcriptomic and epigenomic analyses reveal new regulators of murine brown adipogenesis. PLoS Genet..

[33] Lee J.E., Wang C., Xu S., Cho Y.W., Wang L., Feng X., Baldridge A., Sartorelli V., Zhuang L., Peng W. (2013). H3K4 mono- and di-methyltransferase MLL4 is required for enhancer activation during cell differentiation. eLife.

[34] Lai B., Lee J.E., Jang Y., Wang L., Peng W., Ge K. (2017). MLL3/MLL4 are required for CBP/p300 binding on enhancers and super-enhancer formation in brown adipogenesis. Nucleic Acids Res..

[35] Shi Y., Lan F., Matson C., Mulligan P., Whetstine J.R., Cole P.A., Casero R.A. (2004). Histone demethylation mediated by the nuclear amine oxidase homolog lsd1. Cell.

[36] Zeng X., Jedrychowski M.P., Chen Y., Serag S., Lavery G.G., Gygi S.P., Spiegelman B.M. (2016). Lysine-specific demethylase 1 promotes brown adipose tissue thermogenesis via repressing glucocorticoid activation. Genes Dev..

[37] Roh H.C., Tsai L.T.Y., Shao M., Tenen D., Shen Y., Kumari M., Lyubetskaya A., Jacobs C., Dawes B., Gupta R.K. (2018). Warming induces significant reprogramming of beige, but not brown, adipocyte cellular identity. Cell Metab..

[38] Abe Y., Rozqie R., Matsumura Y., Kawamura T., Nakaki R., Tsurutani Y., Tanimura-Inagaki K., Shiono A., Magoori K., Nakamura K. (2015). JMJD1A is a signal-sensing scaffold that regulates acute chromatin dynamics via SWI/SNF association for thermogenesis. Nat. Commun..

[39] Tateishi K., Okada Y., Kallin E.M., Zhang Y. (2009). Role of Jhdm2a in regulating metabolic gene expression and obesity resistance. Nature.

[40] Loft A., Forss I., Siersbaek M.S., Schmidt S.F., Larsen A.S., Madsen J.G., Pisani D.F., Nielsen R., Aagaard M.M., Mathison A. (2015). Browning of human adipocytes requires KLF11 and reprogramming of PPARgamma superenhancers. Genes Dev..

[41] Waki H., Nakamura M., Yamauchi T., Wakabayashi K., Yu J., Hirose-Yotsuya L., Take K., Sun W., Iwabu M., Okada-Iwabu M. (2011). Global mapping of cell type-specific open chromatin by faire-seq reveals the regulatory role of the nfi family in adipocyte differentiation. PLoS Genet..

[42] Hiraike Y., Waki H., Yu J., Nakamura M., Miyake K., Nagano G., Nakaki R., Suzuki K., Kobayashi H., Yamamoto S. (2017). NFIA co-localizes with PPARgamma and transcriptionally controls the brown fat gene program. Nat. Cell Biol..

[43] Rajbhandari P., Thomas B.J., Feng A.C., Hong C., Wang J., Vergnes L., Sallam T., Wang B., Sandhu J., Seldin M.M. (2018). IL-10 signaling remodels adipose chromatin architecture to limit thermogenesis and energy expenditure. Cell.

[44] Kiskinis E., Hallberg M., Christian M., Olofsson M., Dilworth S.M., White R., Parker M.G. (2007). RIP140 directs histone and DNA methylation to silence Ucp1 expression in white adipocytes. EMBO J..

[45] Shore A., Karamitri A., Kemp P., Speakman J.R., Lomax M.A. (2010). Role of Ucp1 enhancer methylation and chromatin remodelling in the control of Ucp1 expression in murine adipose tissue. Diabetologia.

[46] Derecka M., Gornicka A., Koralov S.B., Szczepanek K., Morgan M., Raje V., Sisler J., Zhang Q., Otero D., Cichy J. (2012). Tyk2 and Stat3 regulate brown adipose tissue differentiation and obesity. Cell Metab..

[47] Lim Y.C., Chia S.Y., Jin S., Han W., Ding C., Sun L. (2016). Dynamic DNA methylation landscape defines brown and white cell specificity during adipogenesis. Mol. Metab..

[48] Yue F., Cheng Y., Breschi A., Vierstra J., Wu W., Ryba T., Sandstrom R., Ma Z., Davis C., Pope B.D. (2014). A comparative encyclopedia of DNA elements in the mouse genome. Nature.

[49] Park Y.K., Ge K. (2017). Glucocorticoid receptor accelerates, but is dispensable for, adipogenesis. Mol. Cell. Biol..

[50] Roh H.C., Tsai L.T., Lyubetskaya A., Tenen D., Kumari M., Rosen E.D. (2017). Simultaneous transcriptional and epigenomic profiling from specific cell types within heterogeneous tissues in vivo. Cell Rep..

[51] Harms M.J., Lim H.W., Ho Y., Shapira S.N., Ishibashi J., Rajakumari S., Steger D.J., Lazar M.A., Won K.J., Seale P. (2015). PRDM16 binds MED1 and controls chromatin architecture to determine a brown fat transcriptional program. Genes Dev..

[52] Pradhan R.N., Bues J.J., Gardeux V., Schwalie P.C., Alpern D., Chen W., Russeil J., Raghav S.K., Deplancke B. (2017). Dissecting the brown adipogenic regulatory network using integrative genomics. Sci. Rep..

[53] Kurdistani S.K., Grunstein M. (2003). Histone acetylation and deacetylation in yeast. Nat. Rev. Mol. Cell Biol..

[54] Shahbazian M.D., Grunstein M. (2007). Functions of site-specific histone acetylation and deacetylation. Annu. Rev. Biochem..

[55] Bannister A.J., Kouzarides T. (2011). Regulation of chromatin by histone modifications. Cell Res..

[56] Jenuwein T., Allis C.D. (2001). Translating the histone code. Science.

[57] Owen D.J., Ornaghi P., Yang J.C., Lowe N., Evans P.R., Ballario P., Neuhaus D., Filetici P., Travers A.A. (2000). The structural basis for the recognition of acetylated histone H4 by the bromodomain of histone acetyltransferase gcn5p. EMBO J..

[58] Nielsen P.R., Nietlispach D., Mott H.R., Callaghan J., Bannister A., Kouzarides T., Murzin A.G., Murzina N.V., Laue E.D. (2002). Structure of the HP1 chromodomain bound to histone H3 methylated at lysine 9. Nature.

[59] Jacobs S.A., Khorasanizadeh S. (2002). Structure of HP1 chromodomain bound to a lysine 9-methylated histone H3 tail. Science.

[60] Bannister A.J., Zegerman P., Partridge J.F., Miska E.A., Thomas J.O., Allshire R.C., Kouzarides T. (2001). Selective recognition of methylated lysine 9 on histone H3 by the HP1 chromo domain. Nature.

[61] Duteil D., Tosic M., Lausecker F., Nenseth H.Z., Muller J.M., Urban S., Willmann D., Petroll K., Messaddeq N., Arrigoni L. (2016). Lsd1 ablation triggers metabolic reprogramming of brown adipose tissue. Cell Rep..

[62] Abe Y., Fujiwara Y., Takahashi H., Matsumura Y., Sawada T., Jiang S., Nakaki R., Uchida A., Nagao N., Naito M. (2018). Histone demethylase JMJD1A coordinates acute and chronic adaptation to cold stress via thermogenic phospho-switch. Nat. Commun..

[63] Loven J., Hoke H.A., Lin C.Y., Lau A., Orlando D.A., Vakoc C.R., Bradner J.E., Lee T.I., Young R.A. (2013). Selective inhibition of tumor oncogenes by disruption of super-enhancers. Cell.

[64] Roe J.S., Mercan F., Rivera K., Pappin D.J., Vakoc C.R. (2015). Bet bromodomain inhibition suppresses the function of hematopoietic transcription factors in acute myeloid leukemia. Mol. Cell.

[65] Lee J.E., Park Y.K., Park S., Jang Y., Waring N., Dey A., Ozato K., Lai B., Peng W., Ge K. (2017). Brd4 binds to active enhancers to control cell identity gene induction in adipogenesis and myogenesis. Nat. Commun..

[66] Rajakumari S., Wu J., Ishibashi J., Lim H.W., Giang A.H., Won K.J., Reed R.R., Seale P. (2013). Ebf2 determines and maintains brown adipocyte identity. Cell Metab..

[67] Whyte W.A., Orlando D.A., Hnisz D., Abraham B.J., Lin C.Y., Kagey M.H., Rahl P.B., Lee T.I., Young R.A. (2013). Master transcription factors and mediator establish super-enhancers at key cell identity genes. Cell.

[68] Sim C.K., Kim S.Y., Brunmeir R., Zhang Q., Li H., Dharmasegaran D., Leong C., Lim Y.Y., Han W., Xu F. (2017). Regulation of white and brown adipocyte differentiation by RhoGAP DLC1. PLoS ONE.

[69] Ng R., Hussain N.A., Zhang Q., Chang C., Li H., Fu Y., Cao L., Han W., Stunkel W., Xu F. (2017). Mirna-32 drives brown fat thermogenesis and trans-activates subcutaneous white fat browning in mice. Cell Rep..

[70] Smemo S., Tena J.J., Kim K.H., Gamazon E.R., Sakabe N.J., Gomez-Marin C., Aneas I., Credidio F.L., Sobreira D.R., Wasserman N.F. (2014). Obesity-associated variants within *FTO* form long-range functional connections with *IRX3*. Nature.

[71] Claussnitzer M., Dankel S.N., Kim K.H., Quon G., Meuleman W., Haugen C., Glunk V., Sousa I.S., Beaudry J.L., Puviindran V. (2015). Fto obesity variant circuitry and adipocyte browning in humans. N. Engl. J. Med..

[72] Ng Y., Tan S.X., Chia S.Y., Tan H.Y., Gun S.Y., Sun L., Hong W., Han W. (2017). Hoxc10 suppresses browning of white adipose tissues. Exp. Mol. Med..

[73] Kang S., Tsai L.T., Zhou Y., Evertts A., Xu S., Griffin M.J., Issner R., Whitton H.J., Garcia B.A., Epstein C.B. (2015). Identification of nuclear hormone receptor pathways causing insulin resistance by transcriptional and epigenomic analysis. Nat. Cell Biol..

